# Atrial fibrillation and what else?

**DOI:** 10.1007/s12471-022-01669-9

**Published:** 2022-02-25

**Authors:** S. M. van den Bogaard, F. E. Vervaat, P. H. van der Voort

**Affiliations:** Department of Cardiology, Catharina Hospital, Eindhoven, The Netherlands

## Answer

**Fig. 1:**
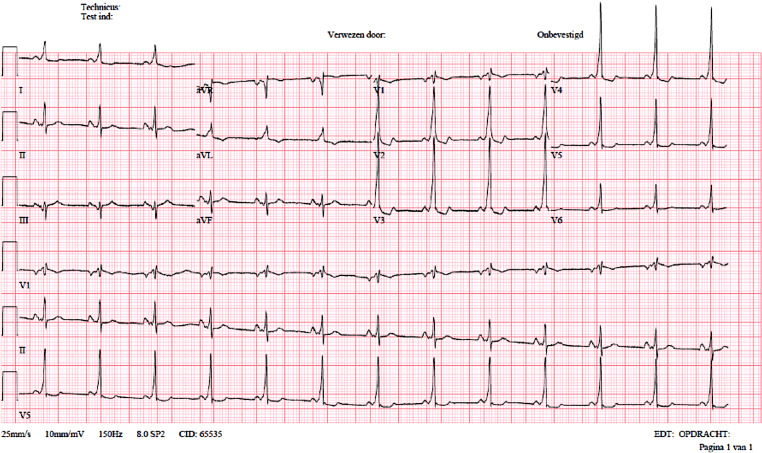
ECG after electrical cardioversion showing sinus rhythm with ventricular pre-excitation. *ECG* electrocardiogram

Initially, the bursts of wide complex tachycardia were diagnosed as non-sustained ventricular tachycardia (VT); however, higher doses of beta blocker increased the occurrence of wide complex tachycardia, without affecting the heart rate in atrial fibrillation (AF). The respiratory condition of the patient deteriorated further during the hospital stay, necessitating transfer to the intensive care unit, intubation and initiation of mechanical ventilation. To optimise the haemodynamic state, transoesophageal echocardiography-guided electrical cardioversion was performed after left atrial appendage thrombosis had been excluded. The electrical cardioversion was successful with restoration of sinus rhythm (Fig. 1).

The electrocardiogram during sinus rhythm now revealed ventricular pre-excitation over a left-sided accessory pathway (AP). The patient’s first electrocardiogram (see Fig. 1a in the Question) had shown AF with exclusively narrow QRS complexes; antegrade conduction over the AP was concealed by retrograde (partial) conduction in the AP. Beta blockers slowed the conduction in the AV node, decreasing retrograde concealment and facilitating antegrade conduction over the AP (see Fig. 1b in the Question) [1].

Beta blockers have been shown to increase ventricular rate [2]. Potential mechanisms include (1) direct shortening of the AP refractory period, which is unlikely [3], (2) indirect shortening of the refractory period by catecholamines and (3) decreased concealed conduction [2]. In the current guidelines, beta blockers are contra-indicated in patients with pre-excitation and AF for this reason. After cardioversion, the patient remained in sinus rhythm, but he died from pulmonary complications of COVID-19.
